# Utilization of a novel patient-specific 3D-printing template for percutaneous endoscopic transforaminal discectomy: results from a randomized controlled trial

**DOI:** 10.3389/fnins.2024.1323262

**Published:** 2024-04-12

**Authors:** Xin Huang, Qipeng Luo, Chen Liang, Yixuan Wang, Donglin Jia, Shuiqing Li, Xiangyang Guo

**Affiliations:** ^1^Department of Pain Medicine, Peking University Third Hospital, Peking University Health Science Center, Beijing, China; ^2^Department of Anesthesiology, Peking University Third Hospital, Peking University Health Science Center, Beijing, China

**Keywords:** lumbar disc herniation, percutaneous endoscopic transforaminal discectomy, 3D printing, patient-specific, individualization, computer-assisted surgery

## Abstract

**Background:**

The learning curve for percutaneous endoscopic transforaminal discectomy (PETD) is steep, especially for the puncturing and localization procedures. The implementation of 3D printing technology may solve this problem.

**Methods:**

A novel individualized 3D-printing template (3D-PT) was designed and utilized in PETD. A prospective randomized controlled trial was performed. A total of 28 patients with lumbar disc herniation treated with PETD were analyzed. Of these, 14 patients were treated with the assistance of 3D printing technology (3D-PT group) in conjunction with fluoroscopy, while the remaining 14 patients were treated exclusively under the guidance of C-arm fluoroscopy (control group).

**Results:**

The number of puncture attempts in the 3D-PT group was significantly less than in the control group (1.36 ± 0.63 vs. 6.07 ± 3.08, *p* = 0.000). The 3D-PT group exhibited a significant reduction in both intraoperative puncture fluoroscopies (2.71 ± 1.27 vs. 12.14 ± 6.15, *p* = 0.000) and the overall number of fluoroscopies (2.71 ± 1.27 vs. 17.43 ± 6.27, *p* = 0.000). In the 3D-PT group, there was a significant reduction in both the puncture time (5.77 ± 1.82 vs. 13.99 ± 4.36, *p* = 0.000) and the total operation time (60.39 ± 9.78 vs. 76.25 ± 17.78, *p* = 0.007). Complications were not observed in either group.

**Conclusion:**

The application of the novel individualized 3D-PT for PETD is effective and safe. The technique has substantial potential and is worth widely promoting.

## Introduction

Percutaneous endoscopic transforaminal discectomy (PETD) has become a popular surgical option for lumbar disc herniations, due to its various advantages such as minimal injury, reliable clinical efficacy, avoidance of general anesthesia, and earlier functional recovery (Pan et al., 2020). However, the learning curve for PETD is steep (Ahn et al., 2020).

**Figure 1:**
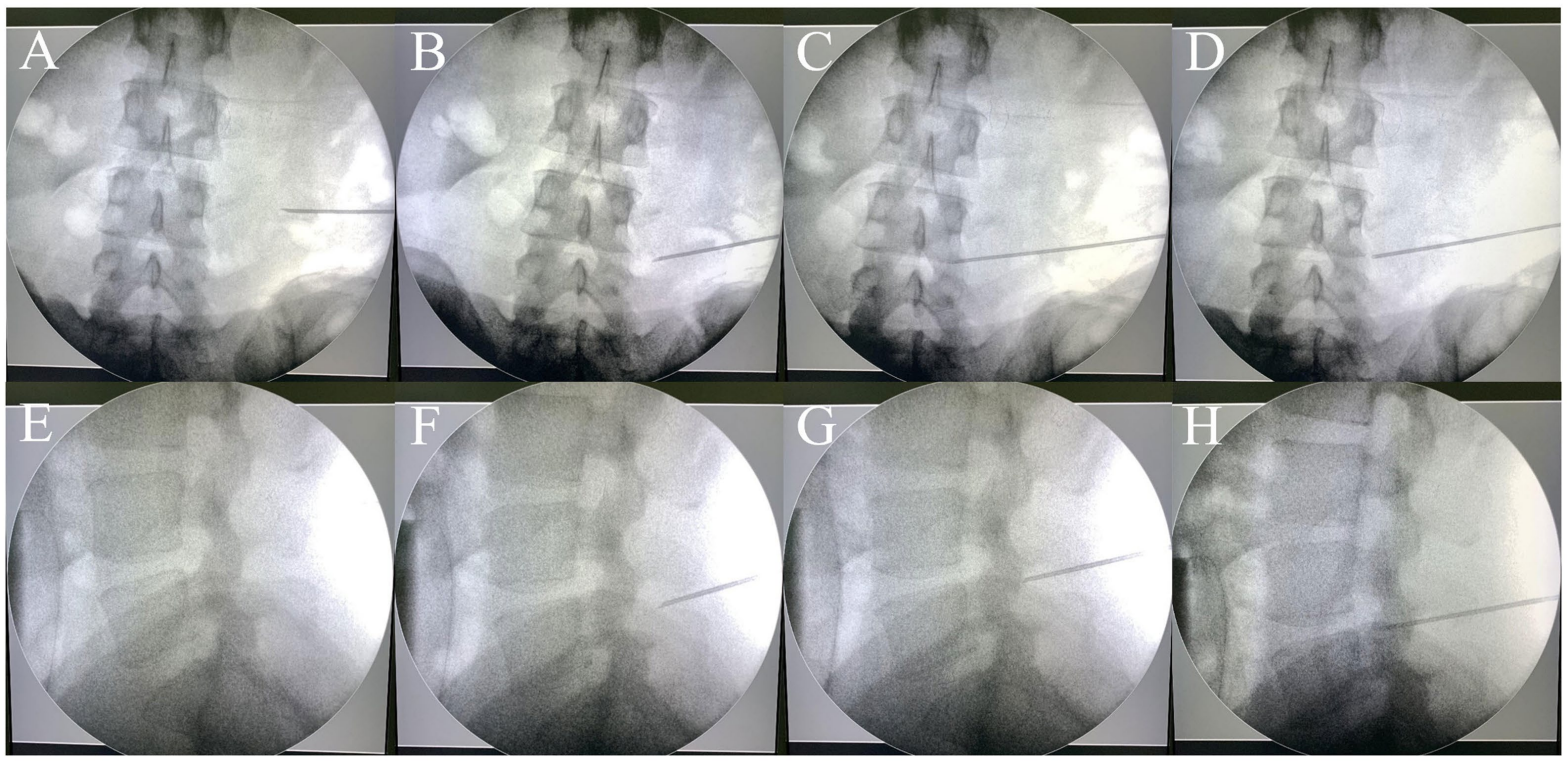
The traditional puncture process requires continuous adjustments under the guidance of a C-arm fluoroscopy. Repeated adjustments of the needle **(A–C, E–G)** were conducted until the target was reached **(D,H)**. **(A–D)**: anteroposterior views; **(E–H)**: lateral views.

Compared to traditional posterior surgery, PETD requires a specialized puncture procedure from the skin entrance point to the intervertebral foramen within the body. As the skin entry point of PETD is usually more external, it can be difficult to position and target. The puncturing process demands extreme caution because incorrect punctures can result in obstructions by abnormal bony structures (such as high iliac crests) (Tezuka et al., 2017), and damage to nerves (Choi et al., 2013), blood vessels (Kim et al., 2009), and abdominal organs (Ahn, 2012). Currently, the surgeons need to place the needle according to their previous experience under the guidance of C-arm fluoroscopy. If the position of the needle is not satisfactory, repeated adjustments of the needle will be performed until it reaches the target (Figure 1). For beginners and in some difficult situations, the puncture location needs to be adjusted dozens of times. Excessive adjustments can lead to an extension of the surgery time, an increase in the number of punctures, and an increase in the number of fluoroscopic views, thereby increasing the potential risk of radiation damage (Yu and Khan, 2014; Iprenburg et al., 2016).

To address these challenges, a novel individualized 3D-printing template (3D-PT) has been developed. Through CT reconstruction and preoperative planning, an accurate puncture trajectory was designed. The application of 3D-PT can assist surgeons in finding the appropriate puncture point and trajectory, thereby simplifying the puncture process. We conducted a prospective randomized controlled trial, and the clinical efficacy and safety of 3D-PT were evaluated.

## Materials and methods

### Patient characteristics

The study protocol was approved by the local institutional review board (2022-427-02) and registered at ClinicalTrials.gov (ID: NCT05632835). All patients signed an informed consent document. Between November 2022 and December 2023, 28 patients with lumbar disc herniation were treated with PETD. Patients were randomized into two groups using a random number table and the randomization grouping scheme was deployed to the REDCap platform from the National Research Institute of Health and Big Data of Peking University to achieve centralized concealment of the randomization grouping scheme. This study did not use the blinding method. Of these, 14 patients were treated with the guidance of 3D-PT (3D-PT group), and the remaining 14 patients were treated under the guidance of C-arm fluoroscopy (control group). Patient characteristics are listed in Table 1.

**Table 1:** Patient characteristics.

Characteristics	3D-PT Group	Control Group	*p*-values
Age (years)	49.07 ± 21.55	42.86 ± 17.64	0.411
Gender			0.706
Male	6	8	
Female	8	6	
BMI (kg/m2)	24.58 ± 1.787	23.48 ± 2.15	0.153
Operative level			1
L3/4	2	1	
L4/5	9	10	
L5/S1	3	3	

BMI, body mass index; 3D-PT, 3D-printing template.

The inclusion criterion was the presence of lumbar disc herniation, verified through MRI imaging, and characterized by symptoms such as radicular pain or a progressive neurologic deficit unresponsive to conservative therapies. The exclusion criteria included segmental instability, pregnancy, infection, bleeding disorder, and refusal by the patient.

### CT scanning and preoperative planning

**Figure 2:**
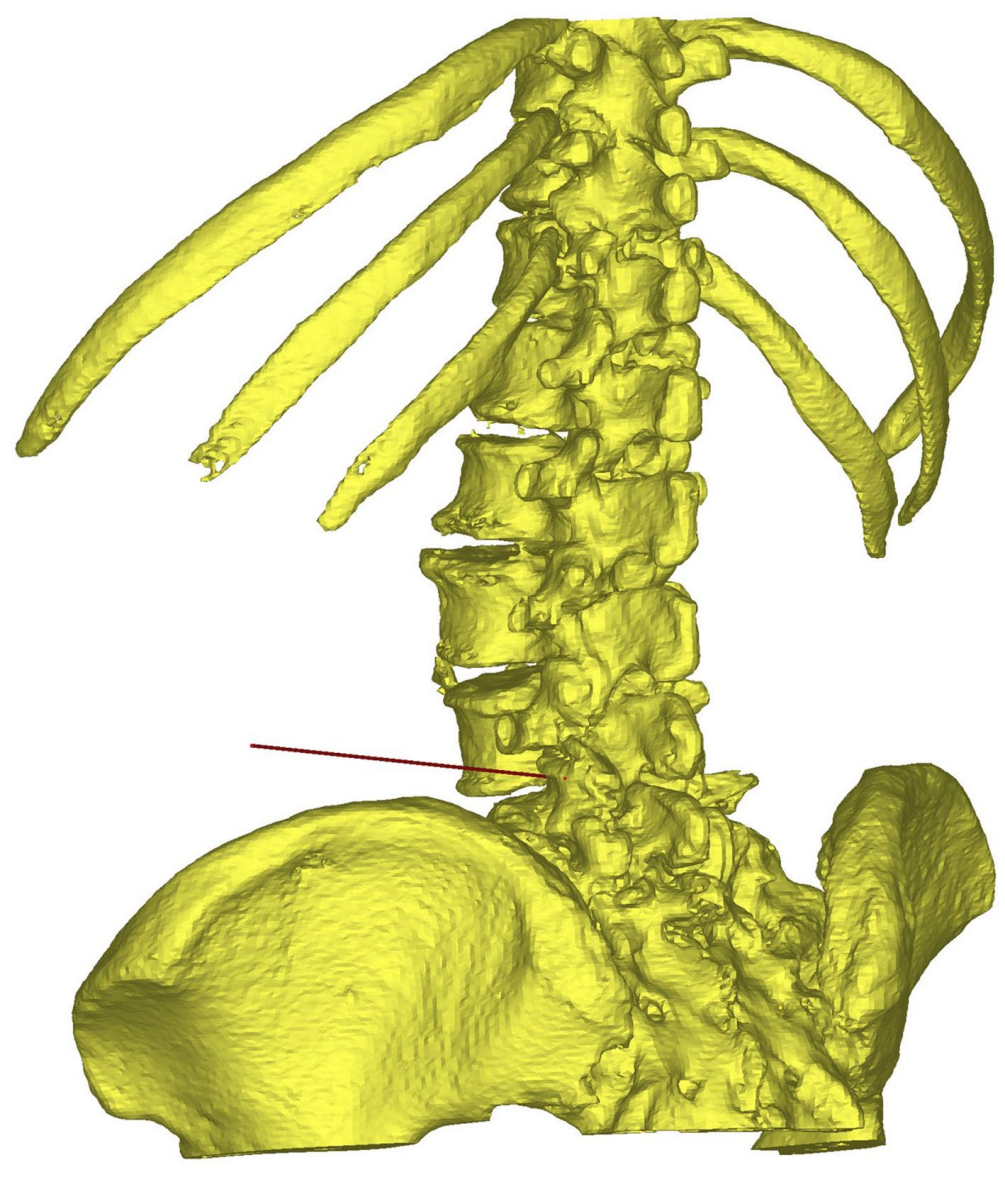
The puncture trajectory for percutaneous endoscopic transforaminal discec-tomy was designed on the reconstructed 3D model.

All patients were subjected to computed tomography (CT) scans two days before the surgery. In the 3D-PT group, the patient was positioned in the prone position, supported by chest pillows and pelvic pads during the CT imaging procedure. The skin of each patient’s lumbar region was exposed. The approximate location of the skin entrance point was deduced based on the target segment. Three metal electrodes, serving as localization markers, were placed at three distinct sites medial to the skin entrance point area. The position of the electrodes should be within the area covered by the guide plate, but should not overlap with the puncture site area; their positions were delineated on the skin with a marker pen. Subsequently, a thin-slice lumbar CT scan was conducted. The Digital Imaging and Communications in Medicine (DICOM) data were acquired and imported into the Mimics 20.0 software (Materialise, Leuven, Belgium). The 3D models of the spine, skin, and metal electrodes were reconstructed (Hu et al., 2021; Wu et al., 2023). The target area, specifically the ventral aspect of the superior articular process, was marked. A suitable puncture trajectory was meticulously delineated (Figure 2). The puncture distance from the skin entrance point to the target area was precisely measured.

### Preparation of individualized 3D-printing template

**Figure 3:**
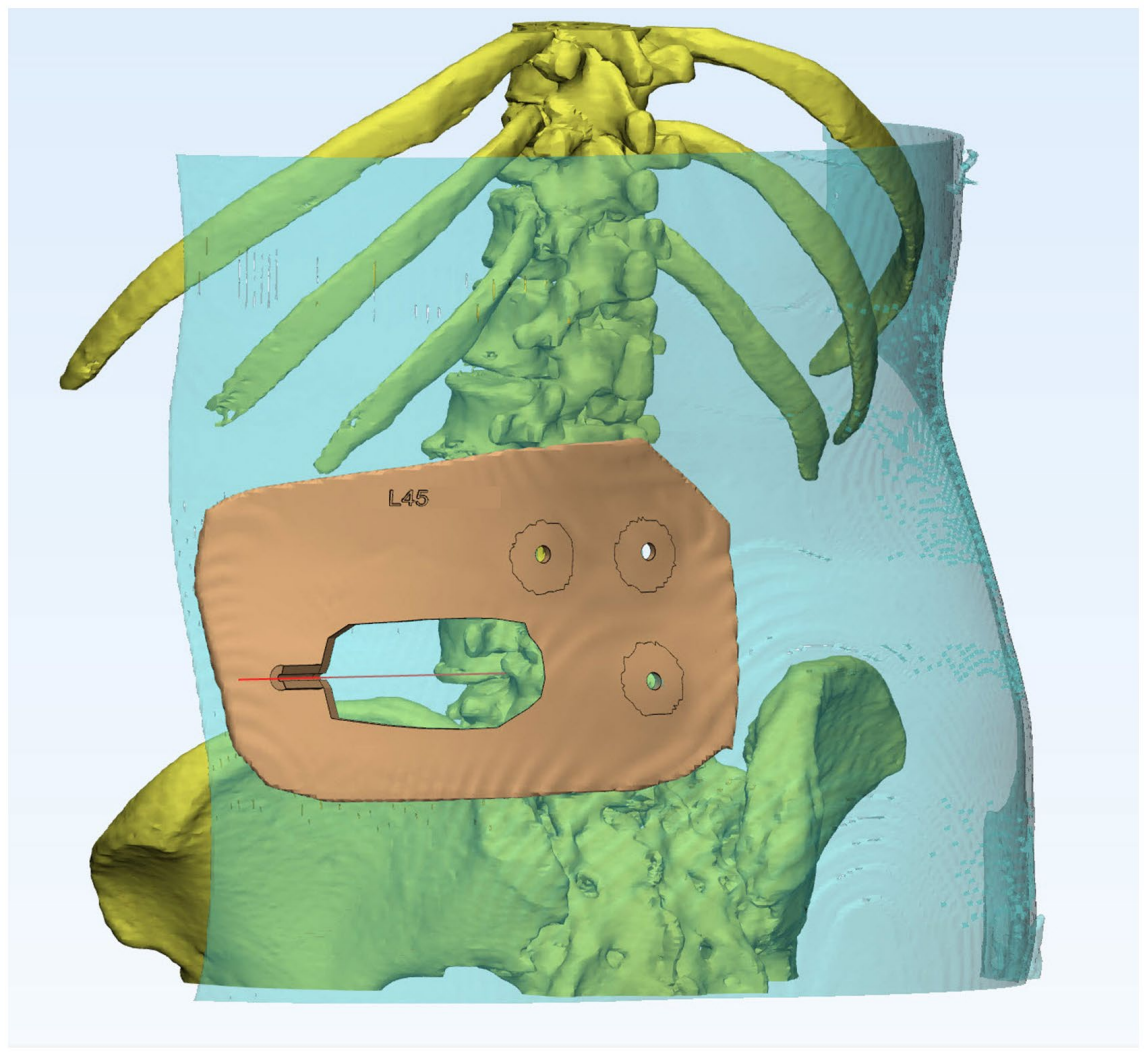
Individualized 3D-printing template with a base plate based on the contour of the skin, three localization holes, and a semi-circular tubular puncture channel.

**Figure 4:**
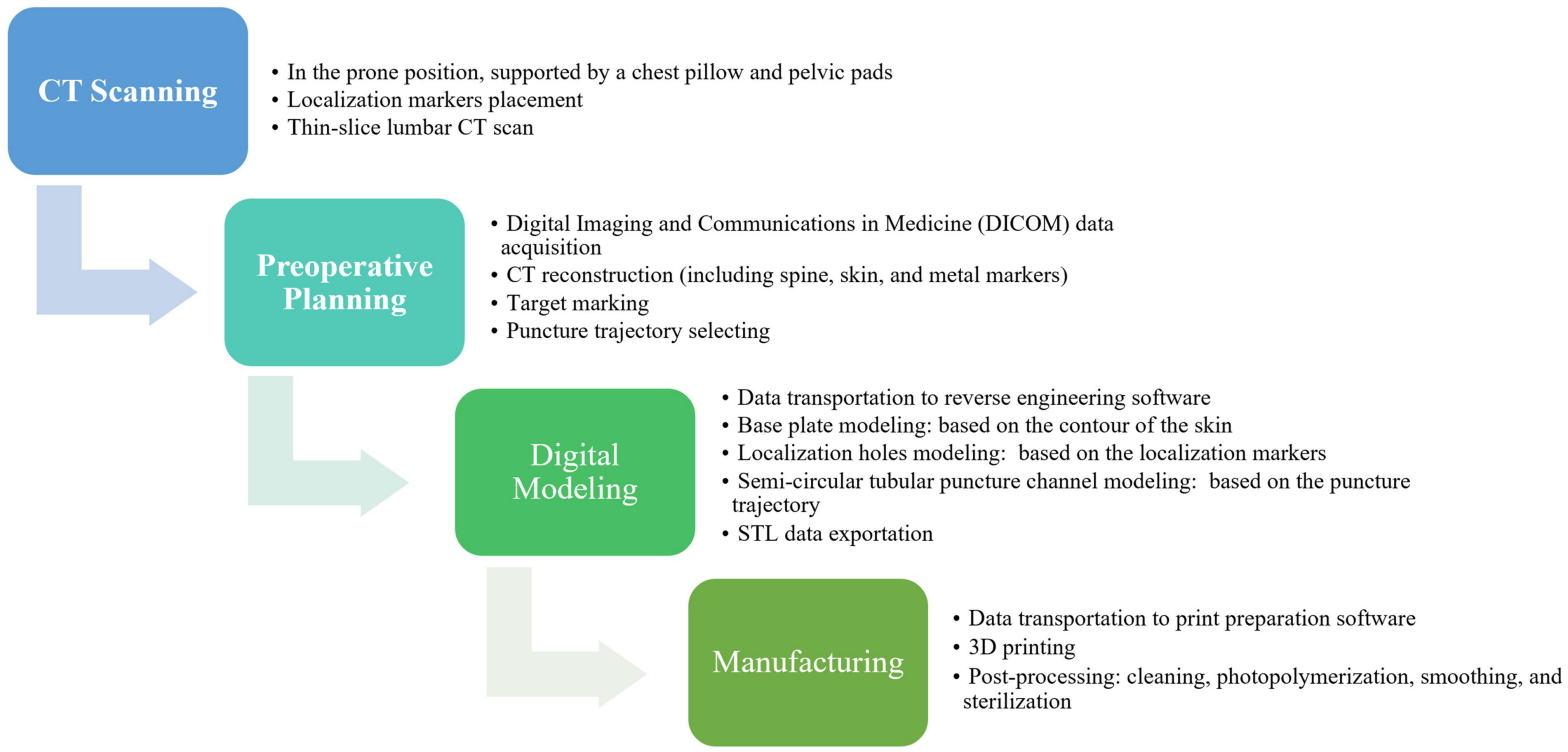
The production processes of individualized 3D-printing template.

The data of the spine, skin, metal electrodes, and puncture trajectory were then imported into 3-matic software (Materialise, Leuven, Belgium) for digital modeling of individualized 3D-PT (Figure 3; Hu et al., 2021). Based on the contour of the skin and the puncture trajectory, a guide template with a thickness of 3 mm was constructed (Figure 3). On the base plate of the template, three localization holes with a diameter of 5 mm were created at the positions corresponding to the three metal electrodes. The puncture channel for the 18-gage needle was designed with a semi-circular tubular geometry, featuring a diameter of 2 mm. The medial side of the junction between the puncture channel and the base plate was subjected to hollowing. The optimized Standard Template Library (STL) data of the 3D-PT was exported to PreForm print preparation software (Formlabs, Boston, United States), medical-grade photosensitive resin (Formlabs, Boston, United States) was used and was finally printed using Form 3+ 3D rapid-forming printer (Form-labs, Boston, United States). Subsequently, the 3D-PT was cleaned, photopolymerized, smoothed, sterilized using ethylene oxide, and reserved for intraoperative use. It often takes 8–12 h to complete the printing process. The manufacturing process for the individualized 3D-printing template is shown in Figure 4.

### Localization and puncture procedure

**Figure 5:**
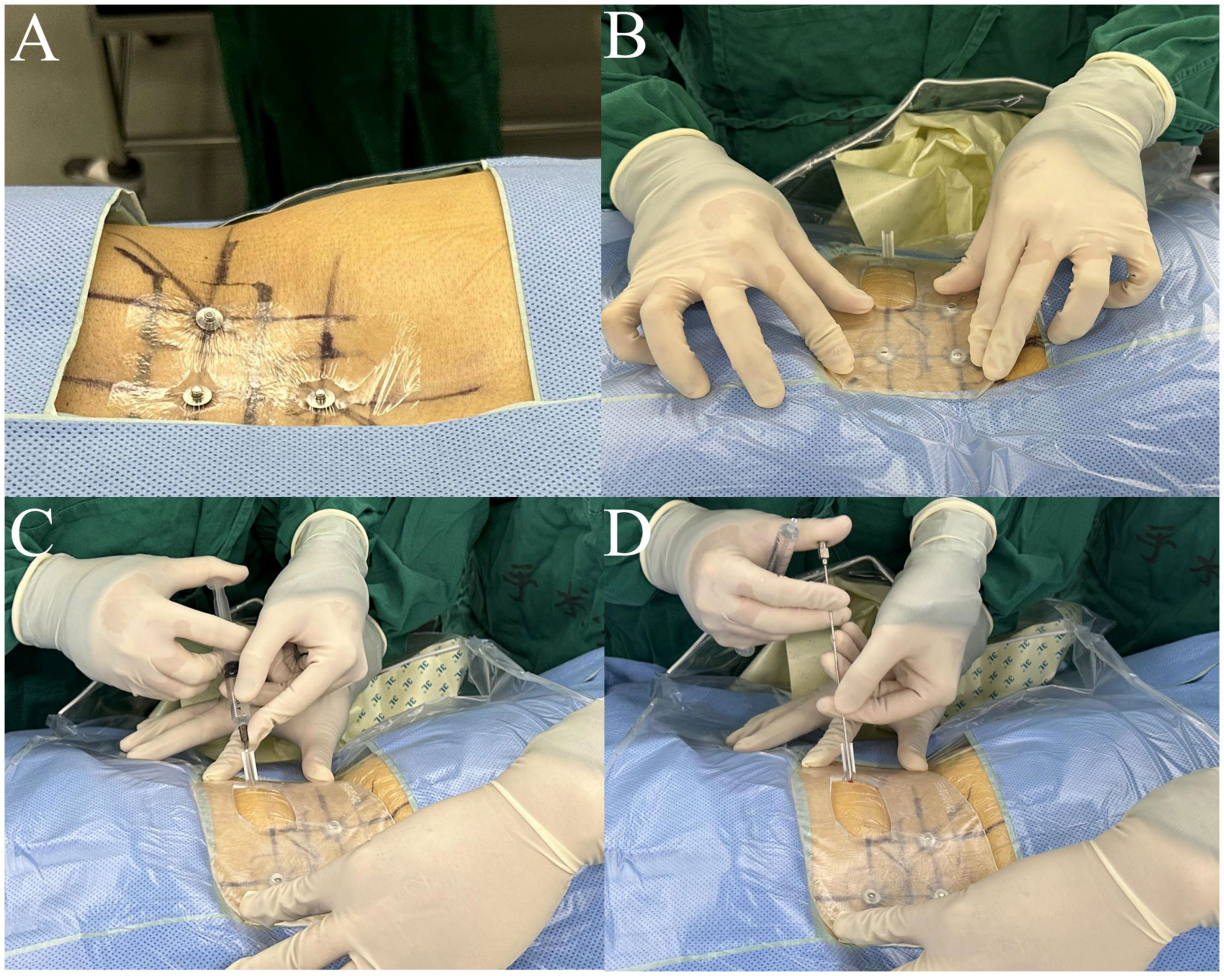
Intraoperative application of the 3D-printing template. **(A)** Three sterile metal electrodes were placed at the localization points; **(B)** the sterilized 3D-PT was anchored to the three metal electrodes adhering to the skin closely; **(C)** local anesthesia was ad-ministered at the skin entry point; **(D)** a puncture needle was put into the semi-circular guide rod, following the direction of the puncture channel.

In the 3D-PT group, the patient was positioned in a prone position, supported by chest pillows and a pelvic pad. The skin in the lumbar region was exposed, subsequently disinfected, and draped. Three sterile metal electrodes were positioned at the locations marked during the preoperative CT scan and secured using adhesive films (Figure 5A). The sterilized 3D-PT was appropriately positioned on the patient’s back, closely adhering to the skin surface. The three localization holes on the 3D-PT were anchored to the three metal electrodes on the patient’s body surface (Figure 5B). Local anesthesia was administered at the skin entry point, as indicated by the 3D-PT (Figure 5C). An 18-gauge puncture needle was introduced into the semi-circular guide rod, adhering to the direction outlined by the puncture channel (Figure 5D). The puncture distance was determined based on the preoperative plan. Subsequently, anteroposterior (AP) and lateral radiographs were conducted to confirm the location and direction of the puncture needle. If the position was unsatisfactory, the 3D-PT was shifted and removed, and further adjustments of the puncture were made under fluoroscopic guidance until reaching the target.

For the control group, preoperative localization was carried out utilizing traditional methods (Yeung and Tsou, 2002). Briefly, Kirschner wires were placed on the patient’s back and lateral sides, and then AP and lateral radiographs were performed to determine the puncture point location. The puncture procedure was performed freehand without a 3D-PT. The puncture needle was adjusted repeatedly under X-ray guidance until it reached the target (Figure 1).

After the puncture procedure, the remaining steps of the PETD were performed on patients in both groups. The number of puncture attempts, the number of fluoros-copies, the puncture time, and the operation time were evaluated. The patients were followed up one-month post-surgery, the post-operation Visual Analog Scale (VAS), the Oswestry Disability Index (ODI), and the rate of complications were evaluated.

### Sample size

To explore the experimental conditions and procedures, we initially included 6 cases in our preliminary pilot. Based on our preliminary pilot data, with α = 0.05 and a test power of 80%. Using the independent samples *t*-test sample size calculation module in PASS software, each group requires 12 cases, and considering a 10% dropout rate, it is preliminarily estimated that each group needs 14 cases.

### Statistical analysis

Experimental data were expressed as mean ± standard deviation (SD). Statistical analysis was performed using the independent sample *t*-test, chi-square test, and Fisher’s exact test. All statistical computations were performed using SPSS software (version 19.0, SPSS Inc., Chicago, IL, United States). A *p*-value of <0.05 in two-tailed tests was considered statistically significant.

## Results

In both the 3D-PT group and the control group, PETD was successfully performed on all patients. The details of intraoperative data are listed in Table 2.

**Table 2:** Comparison of intraoperative records between the two groups.

Characteristics	3D-PT group	Control group	*p*-values
Number of puncture attempts	1.36 ± 0.63	6.07 ± 3.08	0.000
Number of preoperative localization fluoroscopies	0 ± 0	5.29 ± 1.27	0.000
Number of intraoperative puncture fluoroscopies	2.71 ± 1.27	12.14 ± 6.15	0.000
Overall number of fluoroscopies	2.71 ± 1.27	17.43 ± 6.27	0.000
Puncture time	5.77 ± 1.82	13.99 ± 4.36	0.000
Operation time	60.39 ± 9.78	76.25 ± 17.78	0.007
Cases with puncture point adjustment	0	1	1

3D-PT, 3D-printing template.

**Figure 6:**
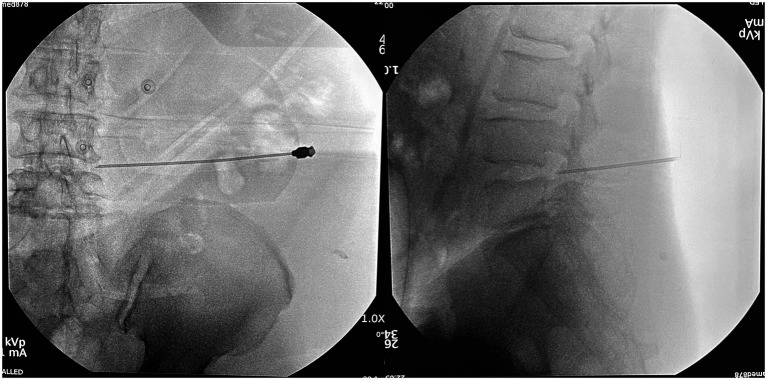
In this case, the patient achieved accurate positioning with only one single puncture.

**Figure 7:**
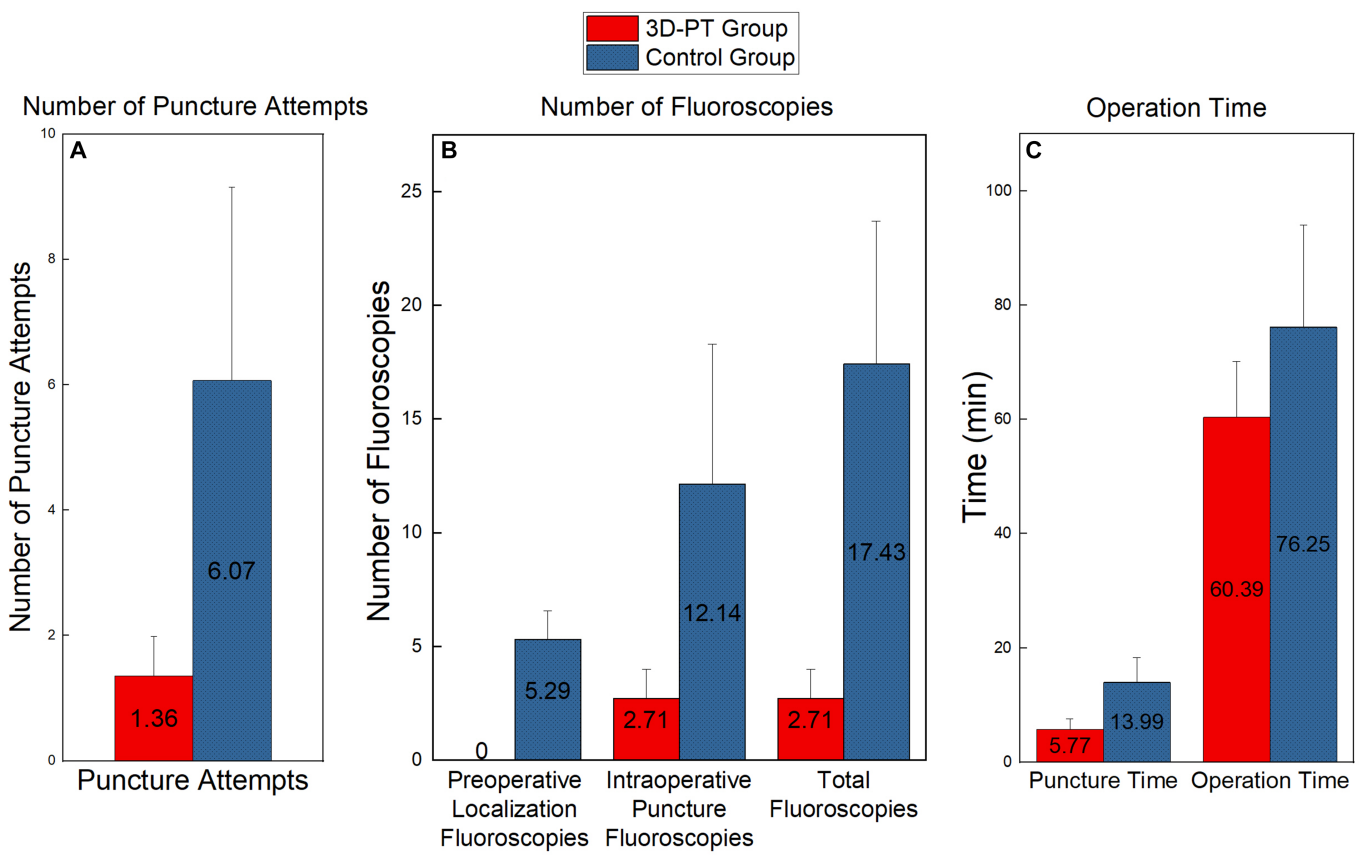
Comparison of the number of puncture attempts **(A)**, the number of fluoros-copies **(B)**, and the operation time **(C)** between the two groups. Significant differences were found in each set of data (*p* < 0.01).

In the 3D-PT group, 10 patients achieved successful targeting with one single puncture (Figure 6), while the remaining 4 required one additional adjustment. The number of puncture attempts is shown in Figure 7A. The number of puncture attempts in the 3D-PT group was significantly less than in the control group (1.36 ± 0.63 vs. 6.07 ± 3.08, *p* = 0.000). Moreover, no patients in the 3D-PT group required adjustments at the puncture point, while one patient in the control group required an adjustment.

The number of fluoroscopies is shown in Figure 7B. For the 3D-PT group, since preoperative localization under C-arm fluoroscopy was not required, the preoperative localization fluoroscopy time was 0. Conversely, the control group had an average of 5.29 ± 1.27 preoperative localization fluoroscopies. Furthermore, the 3D-PT group exhibited a significant reduction in both intraoperative puncture fluoroscopies (2.71 ± 1.27 vs. 12.14 ± 6.15, *p* = 0.000) and the overall fluoroscopies (2.71 ± 1.27 vs. 17.43 ± 6.27, *p* = 0.000) compared to the control group.

The operation time is shown in Figure 7C. In the 3D-PT group, both the puncture time (5.77 ± 1.82 vs. 13.99 ± 4.36, p = 0.000) and the overall operation time (60.39 ± 9.78 vs. 76.25 ± 17.78, *p* = 0.007) were significantly shorter compared to the control group.

Patients in both groups showed VAS and ODI improvement at 1 month postoperatively. The postoperative follow-up data are listed in Table 3. There was no statistical difference in the VAS and ODI between the two groups 1 month postoperatively. Complications such as nerve injury, hematoma, and infection were not observed in the other group.

**Table 3:** Comparison of VAS, ODI, and complication rate between the two groups.

Characteristics	3D-PT group	Control group	*p*-values
Preoperative VAS	6.36 ± 0.93	6.43 ± 1.45	0.878
Postoperative VAS	1.07 ± 0.83	1.14 ± 0.77	0.815
VAS improvement rate	83.37% ± 11.54%	81.41% ± 14.01%	0.690
Preoperative ODI	33.79 ± 5.16	32.86 ± 6.57	0.681
Postoperative ODI	8.57 ± 4.85	9.93 ± 4.19	0.436
ODI improvement rate	74.96 ± 12.23	69.22 ± 13.72	0.253
Complication rate	0	0	1

VAS, Visual Analog Scale; ODI, Oswestry Disability Index; 3D-PT, 3D-printing template.

## Discussion

Puncture and localization are critical steps in minimally invasive spinal surgery, particularly in procedures like PETD. Traditional posterior approach surgery approaches the vertebral plate straight from the back, where the pathway is usually perpendicular to the skin surface, thus lowering the difficulty of puncture. However, the puncture and localization procedures in PETD are difficult, especially for beginners. There are several reasons: (1) a more lateral puncture point: compared with the posterior approach surgery, the skin entrance point for PETD is more lateral (approximately 9–13 cm from the midline), and the puncture angle is more inclined, which increases the difficulty in targeting; (2) prone to obstruction by bony structures: if the direction of the puncture is incorrect, it could be obstructed by bony structures, such as the iliac crest and transverse process; (3) potential for iatrogenic injury due to incorrect puncture direction: if the puncture direction is excessively ventral, it may cause injury to the vessels and abdominal organs (Kim et al., 2009; Ahn, 2012), besides, inappropriate puncture direction could also cause damage to the nerve roots (Choi et al., 2013); (4) abnormal anatomical conditions: the presence of some abnormal anatomical situations, such as high iliac crest (Tezuka et al., 2017), spinal scoliosis (Kim et al., 2015), and post-lumbar internal fixation surgery (Wu et al., 2017), might make the puncture even more challenging. Due to these difficulties, the learning curve for PETD is generally steep (Hsu et al., 2013). Centro Médico Teknon et al. reported that a learning curve of 72 cases was required to achieve the aim of 90% good/excellent results for PETD (Morgenstern et al., 2007).

Currently, the puncture procedure in PETD is performed under the guidance of a C-arm. The AP and lateral radiographic views can be utilized to determine the position of the needle tip: the distance from the needle tip to the midline can be judged from the AP view, while the depth of the puncture needle can be assessed from the lateral view. Based on the position of the needle tip in the X-ray, the puncture needle is repeatedly adjusted manually until it reaches the target site. Repeated adjustments can lead to a series of issues, such as extended surgical time, excessive soft tissue injury due to recurrent punctures, and potential radiation hazards due to excessive fluoros-copy. Iprenburg et al. reported that in PETD, both the surgeon and the patient are likely to be exposed to higher levels of radiation during a surgeon’s early experience (Iprenburg et al., 2016). Navigation and robot-assisted surgical techniques have been implemented clinically to address these issues. However, an additional surgical incision is usually needed for fiducial markers, such as spinous process bone clamps (Wu et al., 2022), which cannot meet the requirement of minimized invasiveness in PETD. Besides, they are not suitable for primary hospitals due to the high costs and complicated requirements (Härtl et al., 2013).

3D printing technology has been found to have considerable application value in the medical field (Mayfield et al., 2022; Moralidou et al., 2022), particularly in Spine Surgery (Sun et al., 2021; Fan et al., 2022). Through 3D printing technology, the operator’s ideas can be incorporated into the 3D printed products. Our previous research has already proposed a 3D method for establishing a precise trajectory in PETD (Huang et al., 2018). In this study, we developed a novel 3D-printed guide plate and the plate has a smaller size, new matching methods, etc., which was different from a previous study (Fan et al., 2022). Through the application of 3D printing guide plates, the preoperatively designed trajectory can be effectively translated into the surgical environment. The 3D-PT contained personalized information including body-surface morphology of the target region, localization holes, and a designated entrance aisle suited for the puncture needle. The novel 3D-printed guide plate has a specialized design. The three localization holes on the base plate were devised for intraoperative anchoring to three metal markers placed on the skin so that the guide plate could be positioned precisely during surgery. The puncture channel for the 18-gauge needle was designed with a semi-circular tubular geometry, and the medial side of the junction between the puncture channel and the base plate was subjected to hollowing. This special configuration permits the lateral removal of the 3D-PT, offering the possibility for subsequent adjustments to the puncture needle without the 3D-PT if deemed necessary.

This kind of 3D-PT technology has a series of advantages, including (1) evolution of the surgical procedure: this transition refines the puncture process from a method characterized by blind and repetitive adjustments to one of precise guidance, potentially attaining pointing accuracy in a single attempt; (2) non-invasive localization: it obviates the need for additional skin incisions to place markers on the skeletal structures, which meets the requirements of minimally invasive spine surgery; (3) significant reduction in puncture attempts: it can markedly diminish the number of puncture attempts, reducing the injuries to soft tissue caused by repetitive punctures; (4) decreased fluoroscopy usage: it significantly reduces the number of intraoperative fluoroscopies, thereby reducing potential radiation risks to both the surgeon and the patient; (5) simplified surgical application: the application during surgery is convenient, requiring no additional complex processes; (6) no need for large-scale equipment: it can be employed in primary hospitals without the need for expensive equipment; (7) simplifies puncture procedure: it can mitigate the difficulty of punctures, especially for some complex situations such as post-lumbar surgery and spinal scoliosis.

The results indicate that the utilization of 3D-PT can markedly reduce the number of punctures. Moreover, the majority of the patients (10/14) in the 3D-PT group managed to achieve accurate placement with one single puncture. Achieving puncture precision in a single attempt eliminates the need for repetitive adjustments during surgery. This not only mitigates the injuries caused by multiple punctures but also decreases the number of fluoroscopies, thereby reducing radiation exposure risks. Moreover, it has the benefit of minimizing surgical time and averting potential complications. In the remaining cases, a one-time successful placement was not realized. However, the initial puncture managed to reach the vicinity of the articular process and it could be subsequently adjusted to the target under the guidance of the C-arm easily. To further enhance the accuracy of usage, several details should be noted: (1) patients should be positioned prone during preoperative CT imaging, to maintain the same posture as during surgery; (2) the metal markers should be accurately placed at the positions marked during the preoperative CT scan; (3) it is necessary to avoid actions such as lumbar bridging that could cause changes in spinal morphology during surgery; (4) the 3D-PT should adhere as closely as possible to the skin without causing significant skin deformation; (5) during puncture, it is imperative to advance accurately along the direction of the guide rod, any deviation in direction could potentially lead to a decrease in precision.

Apart from the reduced number of puncture attempts, the 3D-PT group also exhibited significantly less operation time and fewer fluoroscopies compared to the control group. The reduction in the operation time and fluoroscopies directly correlates with the reduced puncture attempts. In addition to this factor, another significant reason for the reduction in operation time and fluoroscopies is that the 3D-PT group entirely omitted the pre-operative localization phase. With the aid of the 3D-PT, there is no longer a need to identify skin entry points through the placement of Kirschner wires and subsequent fluoroscopy, while the control group had to undergo the process repeatedly to find the appropriate skin entry points. Consequently, there was a significant reduction in the overall operation time and the total number of fluoroscopies in the 3D-PT group.

Besides achieving a significant effect, the results show that the application of 3D-PT during surgery is highly safe. Complications were not observed in either group. Moreover, patients in both groups experienced good relief postoperatively. To further enhance the safety of the 3D printed guide plates, several precautions should be observed: (1) during puncture, it is important to consider the guide plate as a tool only to provide a reference for the puncture entry point and direction, the surgeon should still observe the direction of the puncture and judge the appropriateness of the puncture direction; (2) during surgery, the use of the C-arm should not be completely abandoned, it can still be utilized to confirm the direction and position of the puncture needle if needed; (3) while performing the puncture, it is essential to inquire about the patient’s lower limb sensations, if radiating pain in the lower limbs occurs, it indicates the need to retract and adjust the direction promptly; (4) in cases of noticeable anomalies, the guide plate should be removed promptly to make adjustments under the guidance of the C-arm.

Implementing the above details can enhance the precision and safety of utilizing 3D-PT in PETD. The 3D-PT group achieved favorable results, including fewer puncture attempts, reduced puncture time and operation time, and a fewer number of fluoros-copies, while ensuring good post-operative outcomes. Nonetheless, this study has certain limitations: the sample size is small, and the study is single-center research, a multi-center study is needed for further validation.

## Conclusion

In this study, a novel 3D-PT was developed. After CT reconstruction and pre-operative trajectory planning, an individualized 3D-PT was designed and prepared. The 3D-PT was successfully applied intraoperatively to assist surgeons in finding the appropriate puncture point and trajectory. The application of this innovative 3D-PT can not only reduce the number of puncture attempts and the number of fluoroscopies but also shorten the puncture time and the operation time, while ensuring good post-operative outcomes and safety. Besides, the utilization of 3D-PT is effective and convenient. The technique has substantial potential and is worth widely promoting.

## Data availability statement

The raw data supporting the conclusions of this article will be made available by the authors, without undue reservation.

## Ethics statement

The studies involving humans were approved by the local institutional review board of Peking University Third Hospital. The studies were conducted in accordance with the local legislation and institutional requirements. The participants provided their written informed consent to participate in this study.

## Author contributions

XH: Conceptualization, Data curation, Formal analysis, Investigation, Methodology, Writing – original draft, Writing – review & editing. QL: Conceptualization, Data curation, Methodology, Writing – review & editing. CL: Data curation, Investigation, Methodology, Writing – review & editing. YW: Data curation, Investigation, Writing – review & editing. DJ: Methodology, Writing – review & editing. SL: Conceptualization, Funding acquisition, Methodology, Writing – review & editing. XG: Conceptualization, Funding acquisition, Methodology, Writing – review & editing.
